# Editorial: Neuromodulating bioactive compounds as potential cognitive therapeutics

**DOI:** 10.3389/fnagi.2023.1143193

**Published:** 2023-02-03

**Authors:** Ashish Kumar, Arpita Konar

**Affiliations:** ^1^Brain Science Research Institute, AriBio Co., Ltd., Seongnam-si, Gyeonggi-do, Republic of Korea; ^2^Institute of Health Sciences, Presidency University, Kolkata, India

**Keywords:** phytochemical, neuroprotection, aging, neurodegeneration, natural compounds, pharmacology

Cognitive capacities, including visuospatial abilities, executive functions, learning, and memory, when compromised greatly, affect the quality of life and are an enormous burden on healthcare and society. They are one of the major causes of hospitalization, nursing care and death worldwide (Murman, 2015; Konar et al., 2016; Tucker-Drob, 2019). Cognitive aging exhibits a wide spectrum of functioning, from normal, age-related changes, to mild cognitive impairment and dementia (Davis et al., 2018; Roheger et al., 2022) and is also comorbid with psychiatric disorders (Sheffield et al., 2018; McCleery and Nuechterlein, 2019; Pijnenborg et al., 2020). These disorders show common features of cognitive impairment, with marked heterogeneity in their clinical profiles. With improvements in diagnostics, healthcare facilities, and life expectancy, individuals diagnosed with cognitive disorders are estimated to grow by 115 million by 2050. Multiple factors contribute to the onset of cognitive deficits and their transition to pathological states, including aging, genetic background, lifestyle behaviors, diet, toxin exposures, hormonal states, socioeconomic profiles, comorbidities of cardiovascular disorders, and diabetes (Harman and Martín, 2020; Yan et al., 2020; Liu et al., 2022).

Despite extensive research, pharmacological interventions only seem to alleviate symptoms, without effectively targeting the pathophysiology of cognitive decline. Further, adverse side effects, constraints of the blood-brain barrier, and bioavailability have compromised their clinical success (Casey et al., 2010; Abeysinghe et al., 2020; Rabinovici, 2021). With the limitations of synthetic drugs, bioactive natural compounds may hold great promise as therapeutic leads, owing to their prolonged holistic action and minimal toxicity, thus qualifying as preventive as well as recovery measures. Preclinical and clinical studies have reported a tremendous number of bioactive compounds with neurotrophic and neuroprotective potentials and targeting biological pathways integral to cognition.

Although a substantial number of neuromodulating bioactive compounds with good cognitive efficacies have been identified, there are challenges related to their success in clinical trials. Several hurdles including gaps in scientific validation, knowledge of pharmacokinetics, toxicity, and mechanism of action, which limit the recommendation of these compounds in clinical studies. The scant clinical research available on these compounds is primarily observational and epidemiological and thus they require extensive experimental and interventional approaches to justify their use as cognitive therapeutics. The majority of these bioactive compounds are also limited by rapid metabolism, non-specific targeting, poor solubility, lack of BBB permeability, and reduced bioavailability. Strategies such as targeted modification of compound structures without interrupting biological activity can also improve the bioavailability. Nanoparticle-guided drug delivery is also an emerging approach that can enhance the bioavailability.

The collection of articles published in the Research Topic titled: “*Neuromodulating bioactive compounds as potential cognitive therapeutics*” highlights the neuroprotective roles of various bioactive compounds in diverse preclinical model systems, and, more importantly, focuses on their cellular and molecular targets, both genetic and epigenetic, as well as approaches to increase the bioavailability. Moreover, current drug development strategies, based on modern approaches such as virtual screening and network pharmacology, have also been discussed. The goal of this collection is to stimulate interest and research on neuromodulating bioactive compounds for effective drug development for holistic therapy of cognitive disorders.

In this regard, Singh et al. emphasized that oxidative stress is one of the major contributors to aging and age-related neurodegenerative diseases. The generation of intracellular free radicals is known to affect metabolic functions by biochemical and physiological alterations in the brain, leading to DNA damage and epigenetic modifications, lipid peroxidation, impaired mitochondrial functions, protein misfolding, synaptic and neuronal loss, and ultimately, memory impairment. Natural bioactive compounds, including phytochemical and dietary supplements with inherent high nutritional value and antioxidant properties, have been used in the management of oxidative stress for therapeutic intervention and to ameliorate the deleterious effects of the aging brain. Singh et al. reviewed the antioxidant and memory enhancing properties of plant-derived polyphenols such as flavonoids, phenolic acids, stilbenes, and lignans, and non-phenolic compounds like bacoside-A, withaferin-A, withanolide-A ginkgolide-B, and bilobalide, which are widely used in traditional medicine systems. Since many bioactive compounds have limited ability to cross the blood-brain barrier, nano-herb conjugates may overcome this challenge, helping to improve permeability in the brain to attenuate oxidative stress in an effective manner. In-depth studies and more clinical trials are needed to determine the viability of bioactive compounds in therapeutics.

Li et al. studied the neuroprotective role of a carotenoid active phytochemical, namely, Zeaxanthin, which is present in vegetables and fruits. In previous studies, Zeaxanthin has been reported to protect age-associated cognitive impairment, the risk of atherosclerosis, and Alzheimer's disease due to its anti-oxidant, anti-inflammation, and anti-amyloidogenic properties. Li et al. showed that a daily administration of 60 mg/kg Zeaxanthin for 14 days *via* the intra-gastric route restored the body redox state, as indicated by the glutathione level and the glutathione/glutathione disulfide ratio in the serum of Aβ1–42-treated rats. Zeaxanthin also decreased the Aβ plasma levels and endothelin-1 proteins. Analysis of Zeaxanthin treatment showed a reduction in the receptor for advanced glycation end products, LDL receptor-related protein-1, and inflammatory cytokine interleukin-1 levels in the Aβ1–42-treated rat brains. Behavioral analysis revealed that Zeaxanthin prevents learning and memory deficits in Aβ1–42-treated rats. Morphological analysis using transmission electron microscopy showed that Zeaxanthin may also maintain cerebrovascular endothelial cell architectures in the brain. The study suggests that Zeaxanthin exerts neuroprotection *via* its antioxidant and anti-inflammatory effects on the cerebrovascular tissue by regulating Aβ transportation.

Bhandari et al. studied the effect of *Tinospora cordifolia* dietary supplementation in acyclic aged female rats with a high fat diet. Previously, it has been reported that menopause is responsible for multiple metabolic changes such as dyslipidemia and enhanced adiposity, leading to behavioral alterations including cognitive decline. Hormone replacement therapy has not been effective and it sometimes showed detrimental effects on memory functions. Hormone replacement therapy may also lead to coronary heart diseases, pulmonary embolism, and breast cancer. Bhandari et al. reported that dietary supplementation using stem powder of *T. cordifolia* for 12 weeks improved the learning and memory behavior in high fat diet fed acyclic aged female rats. Molecular analysis of the glial marker GFAP and the microglial protein Iba1 showed a significant decline in the expressions of these proteins, indicating a reduction of neuroinflammation in the hippocampus and the prefrontal cortex of *T. cordifolia–*supplemented rats, compared with high fat diet fed acyclic aged female rats. Further, analysis of the anti-apoptotic proteins AP-1 and Bcl-xL and the pro-apoptotic marker p-BAD showed a significant modulation in the expressions of these proteins, suggesting the decreased apoptotic and pro cell survival effects of *T. cordifolia* supplement in the hippocampus and the prefrontal cortex of high fat diet fed acyclic aged female rats. Moreover, *T. cordifolia* supplement restored the expression of neurotrophic BDNF and Trkβ in the hippocampus and the prefrontal cortex of the high fat diet fed acyclic aged female rats. The encouraging outcomes of this study may pave a path for further exploration of *T. cordifolia* supplement in future studies.

Singh and Paramanik discussed phytoestrogens as potential neuroprotective agents. It is well known that the estrogen hormone and its receptors are important in learning and memory *via* epigenetic regulation of gene expression. A reduction in estrogen signaling is a risk for age-related memory decline and neurodegenerative disorders. Singh and Paramanik reviewed the therapeutic potential of phytoestrogens in memory restoration in aging and different neurological disorders. Besides their antioxidative, anti-apoptotic, and anti-inflammatory actions, phytoestrogens may also epigenetically regulate the expressions of genes involved in memory functions. Phytoestrogen compounds such as resveratrol, genistein, daidzein, and secoisolariciresinol showed neuroprotective, neurogenic, and memory restoration potential in aged estropause female rodents, AD models, and human subjects. Few studies have shown that phytoestrogens may modulate the epigenetic status of chromatin, and, thereby, regulate the expression of genes involved in synaptic plasticity. In particular, genistein- and resveratrol-mediated effects on DNMTs, DNA methylation, HDAC, HAT1, histone acetylation, and miRNA 132 have been reported in several studies. However, more studies are warranted to establish phytoestrogens as an alternative to estrogen therapy for the recovery of cognitive functions in aging and associated disorders.

Ye et al. explored a network pharmacology-based approach to understand the therapeutic potential and working mechanism of *Coptidis Rhizoma* in Alzheimer's disease. In order to address the growing need of polypharmacological strategies, network pharmacology has emerged as an effective approach to investigate the potential of neuromodulating compounds and their mechanisms of action in the treatment of cognitive disorders. Ye et al. studied *Coptidis Rhizoma* and its phytochemical components in the treatment of Alzheimer's disease. Oral bioavailability, drug like properties, and blood ingredients were used to analyze the active components of *Coptidis Rhizoma. Nineteen* active compounds were identified to be related to Alzheimer's disease, along with their potential therapeutic targets; i.e., MAPK8, RELA, TGFB1, and STAT3. Further analysis of the interactive network of compounds and their targets revealed their role in antioxidation, apoptosis, glutathione metabolism, and the HIF-1 signaling pathways. Such analysis of *Coptidis Rhizoma* may be crucial to develop multi-step networking strategies.

## Author contributions

AKu: conceptualization, writing, reviewing, and editing. AKo: writing, reviewing, and editing. Both authors contributed to the article and approved the submitted version.
